# Acknowledgement to Reviewers of *Viruses* in 2015

**DOI:** 10.3390/v8010027

**Published:** 2016-01-22

**Authors:** Viruses Editorial Office

**Affiliations:** MDPI AG, Klybeckstrasse 64, CH-4057 Basel, Switzerland; viruses@mdpi.com

The editors of *Viruses* would like to express their sincere gratitude to the following reviewers for assessing manuscripts in 2015.

We greatly appreciate the contribution of expert reviewers, which is crucial to the journal’s editorial decision-making process. Several steps have been taken in 2015 to thank and acknowledge reviewers. Good, timely reviews are rewarded with a discount off their next MDPI publication. By creating an account on the submission system, reviewers can access details of their past reviews, see the comments of other reviewers, and download a letter of acknowledgement for their records. In addition, MDPI has launched a collaboration with Publons, a website that seeks to publicly acknowledge reviewers on a per journal basis. This is all done, of course, within the constraints of reviewer confidentiality. Feedback from reviewers shows that most see their task as a voluntary and mostly unseen work in service to the scientific community. We are grateful to our reviewers for the contribution they make.

Abd-Alla, AdlyHearing, PatrickPeersen, OlveAbdelwhab, E. M.Heckmann, Josef G.Peikert, TobiasAbdul-Careem, Mohamed FaizalHefferon, Kathleen LauraPeloponese Jr., Jean-MarieAbedon, StephenHeldwein, EkaterinaPeng, Chien-fangAbergel, ChantalHelland, Today EmilPérez Ruiz, MercedesAboulata, A. E.Helle, FrancoisPerez-Romero, PilarAbram, MichaelHenderson, AndrewPeterschmitt, MichelAccotto, Gian PaoloHenke, AndreasPetravic, JankaAchazi, KatharinaHenzy, JamiePeyambari, MahtabAdelman, Zachary NHerath, TharanganiPfeffer, SébastienAdler, BarbaraHernalsteens, Jean-PierrePiantino Ferreira, AJAdlhoch, CorneliaHernandez-Alcoceba, RubenPijlman, GrobenAdriaenssens, EvelienHerrero, SalvadorPique, ClaudineAdriana E., KajonHewson, K. A.Pise-Masison, Cynthia A.Aebischer, AndreaHilgenfeld, RolfPlażuk, DamianAghemo, Alessio MicheleHill, AndyPleschka, StephanAguilar-Lemarroy, AdrianaHirsch, Alec J.Plevka, PavelAirenne, KariHirsch, IvanPodhajcer, Osvaldo L.Aires, AlfredoHishiki, TakayukiPoehlmann, StefanAit-Ali, TaharHobman, Tom C.Poelman, RandyAlejo, AlíHobson-Peters, JodyPolacek, CharlottaAlemany, RamonHoeben, RobPoli, GuidoAllander, TobiasHoenen, ThomasPompei, RaffaelloAlmazan, FernandoHolbrook, Michael R.Pope, WelkinAlonso, Marta M.Hondan, TomoyukiPopham, Holly J.R.Altan-Bonnet, NihalHong, Jiann-rueyPopham, Holly J.Alvarado, Julia BéjarHonko, AnnaPossee, RobertAlvarez, Ronald D.Horie, MasayukiPoveda, EvaÁlvarez Sánchez, JulioHorii, TakuroPowis, S. J.Alvisi, GualtieroHorimoto, TaisukePrangishvili, DavidAmadori, M.Hrabal, RichardPrescott, JosephAnanworanich, JintanatHu, JohnPréville, XavierAndersson, BjornHuang, JinghePyeon, DohunAndrei, GracielaHuang, ChienjinQiao, WentaoAndreoletti, LaurentHuemer, Hartwig P.Quackenbush, SandraAngelova, AssiaHufton, Simon E.Quax, TessaAnsaldi, MireilleHughes, AustinQuer, JosepAnton, JosefaHughes, GrantQuinn, Thomas C.Arif, BasilHumbel, BrunoRabkin, Samuel D.Ariza, Maria EugeniaHummel, MaryRacaniello, VincentArnaud, FrederickHunsperger, ElizabethRagonnet-Cronin, ManonArnold, MichelleHust, MichaelRajawat, YogendraAscencio-Ibanez, JoseHutchinson, Edward C.Rakotondrafara, AurelieAsfor, Amin S.Huthoff, HendrikRandall, Troy D.Azab, WalidIbanez, Trino AscenciaRao, Venigalla B.Azzouz, MimounIcenogle, JosephRatner, LeeBagamian, KarounIshikawa, MasayukiRauf, AbdulBaird, NicholasIshima, RiekoRautenschlein, SilkeBajorek, MonikaIto, NaotoRavoet, JorgenBakonyi, TamasIwanaga, MasashiRay, FelixBalfour, HenryIzquierdo-Useros, NuriaRees, CatherineBálint, ÁdámJackson, TerryReid, StevenBalkema-Buschmann, AnneJackson, WilliamReid, St. PatrickBalvers, Rutger K.Jacobs, N.Reid, TonyBanfield, BruceJacobson, StevenRein, AlanBangham, Charles R.M.Jahrling, PeterReperant, LeslieBannert, NorbertJain, PoojaRessing, MaaikeBar-Joseph, MosheJang, Ho BinRevilla, YolandaBaraka, KhaledJehle, JohannesRey, ChrissieBarbu, MagdaJeske, HolgerRicciardi, RobertBarik, SailenJohnson, Reed F.Rice, AndrewBarklis, EricJohnson, NickRichardson, Charles C.Barrett, JohnJolly, ClareRichert-Pöggeler, KatjaBarrett, Alan D. T.Jones, IanRihn, SuzannahBartha, IstvánJones, ClintonRimstad, EspenBartholomay, LyricJones, JeremyRinderer, Thomas E.Bartlett, David L.Jones, KathrynRisatti, Guillermo R.Barton, DavidJourno, ChloéRisco, CristinaBatchu, Ramesh B.Junglen, Sandra Rivas, C.Bateman, Kelly S.Junichiro, YasunagaRobert-Guroff, MarjorieBausch, DanielKadowaki, TatsuhikoRoberts, SallyBeard, PipKaguni, Jon M.Robertson, Erle S.Becker, StephanKaida, AtsushiRobinson, GeneBeemon, KarenKajaste-Rudnitski, AnnaRoden, RichardBelov, GeorgeKalejta, RobertRodriguez, Jose F.Beniac, Daniel R.Kalergis, Alexis M.Rodriguez, LorenaBenichou, GilesKalinke, UlrichRoh, ChanghyunBenschop, KSKamen, Amine A.Roine, ElinaBerenguer, MarinaKamil, JeremyRomalde, Jesús LópezBergman, NickKanda, TatsuoRomanelli, Maria GraziaBergthaler, AndreasKandathil, AbrahamRomanowski, VictorBerkhout, BenKanduc, DarjaRomero-Brey, InésBernard, Hans-ulrichKang, HyojeungRoques, PierreBeziers, AnnieKang, CongbaoRosenberg, HeleneBhardwaj, NeeruKariwa, HiroakiRossetto, Cyprian C.Biegalke, Bonita J.Karlin, David G.Rossi, John J.Billington, CraigKarlsson, ErikRossman, JeremyBlair, CarolKashanchi, FatahRossmann, Michael G.Blasdel, RobertKeil, Günther M.Rota, Paul ABlasdell, KimKennedy, Richard G.Rothenburg, StefanBlass, DieterKesmir, CanRowan, AileenBlitvich, BradKhalifa, Mahmoud ERowlands, David J.Blome, SandraKhan, ArifaRoy, PollyBlondel, BrunoKhan, MazharRubio, LuisBochkov, Yury A.Khan, Zafar K.Ruggli, NicolasBodem, JochenKhatri, MaheshRussell, RodneyBodily, JasonKhromykh, Alexander A.Ryabov, Eugene V.Bogner, ElkeKhromykh, AlexanderRyabova, LyubaBolling, Bethany G.Kielan, MargaretRyckman, BrentBoris-Lawrie, KathleenKikuchi, YutakaRyu, SangryeolBoulanger, PascaleKim, KisoonSaayman, SheenaBowden, Bruce FKim, SoohyunSadeuh-Mba, SergeBrandt, Curtis R.Kimura, TakashiSadiq, S. KashifBrault, AaronKindrachuk, JasonSaelens, XavierBreckpot, KarineKirchhoff, FrankSáiz, MargaritaBressanelli, StéphaneKirkegaard, KarlaSaleh, Maria-CarlaBreyton, CécileKiron, ViswanathSam Zhou, HeshanBriant, LaurenceKirsebom, LeifSambrie, VittorioBridle, Byram W.Kitchen, Scott G.Sandstrom, PaulBriers, YvesKitchen, AndrewSang, YongmingBrighty, DavidKlasse, Per JohanSaphire, EricaBrinkmann, MelanieKlein, ChristianSapp, MartinBrockmeier, Susan L.Klose, ThomasSargueil, BrunoBrooke, Christopher BKlumpp, JochenSattar, SampurnaBrown, JayKlupp, BarbaraSavenkov, EugeneBruessow, HaraldKobayashi, KappeiSawa, HirofumiBuchholz, Christian J.Kok, Chee ChoyScagnolari, CarolinaBuck, ChristopherKootstra, NeeltjeSchaap, IvanBugert, JoachimKorukluoglu, GulaySchaar, Hilde M. Van DerBull, RowenaKousoulas, Konstantin G.Schang, LuisBullitt, EstherKozak, ChristineScheibenbogen, CarmenBüning, HildegardKranzusch, PhilipSchildgen, OliverBurk, Robert D.Krogstad, PaulSchleiss, MarkBurke, DonKryger, PerSchmaljohn, AlanBüttner, MathiasKuhn, RichardSchmelcher, MathiasCaballero, PrimitivoKuhn, JensSchmidt, ManfredCady, KyleKushibiki, ToshihiroSchmidtke, MichaelaCalisher, CharlesKutter, ElizabethSchneider, DirkCambillau, ChristianKvarnheden, AndersSchnell, Matthias J.Campadelli-Fiume, GabrielaLacomme, Dr. ChristopheSchoborg, Robert V.Campbell, EdwardLacroix, JeannineSchöning, CasparCamus-Bouclainville, ChristelleLaethem, Kristel VanSchountz, TonyCandolfi, MarianelaLafon, MoniqueSchröder, MartinaCañizares, CarmenLai, Michael M. C.Schroeder, Declan C.Cao, Yong-changLaimins, LouSchubert, JörgCAO, PengxingLangereis, Martijn A.Schüpbach, JörgCaplen, Natasha J.Langlois, RyanScotch, MatthewCarbonell, AlbertoLaunes, CristianSeeger, ChristophCardoso, Tereza CLavazza, AntonioSekine, KCarfi, AndreaLavergne, AnneSen, AdrishCarpenter, SusanLavigne, RobSeong, Baik LinCasasnovas, José M.Law, MansonSerwer, PhilipCasseb, JorgeLee, Sang-WonSeto, DonaldCen, OsmanLee, Long HuwSevilla, NoemíCenens, WilliamLeGrice, StuartShai, YechielCeyssens, Pieter JanLelli, DavideShechter, DavidChandran, BalaLemasson, IsabelleShelby, Kent S.Chang, JinhongLemay, GuySherer, Nathan M.Chang, Kuan Y.Leng, QibinShi, ZhengLiCharpentier, CharlotteLever, AndrewShisler, JoannaCharrel, RémiLevin, Henry L.Silbart, Lawrence K.Chejanovsky, NorLi, FengSiliciano, Robert F.Chen, BenjaminLi, XuguangSimón, OihaneChen, JasonLiang, ChenSinclair, JohnChen, QiLiang, ZhenglunSinkins, StevenChen, RongLichty, Brian D.Skalka, Anna MarieChen, Li LiLiikanen, LlkkaSkalsky, RebeccaChiu, WahLin, Cheng-WenSkinner, MikeCho, NamhoonLin, RongtuanSlezak, TomChoi, Kyung HLin, Na-ShengSmerdou, ChristianChong, PeleLindberg, A. MichaelSmit, SandraChow, Yen-HungLindemann, DirkSmith, Kellie NChu, DongLindh, MagnusSmith, DonaldChu, JustinLing, Kai-ShuSmith, David W.Chung, AmyLinial, Maxine L.Smith, DarrenCiccone, Nick A.Lippe, RogerSmith, DarciCichero, ElenaLiu, WenSmith, Jason G.Cicin-Sain, LukaLiu, Shan-LuSmith, Robert A.Ciotti, MarcoLiu, DingxiangSmithgall, Thomas E.Clem, RollieLiu, ShuwenSnyder, Christopher M.Clement, JanLiu, Chia-ChyiSobrino, FranciscoClementi, NicolaLiWang, PatriciaSodeik, BeateCoen, Donald M.Loessner, MartinSong, Dae-SubCoffey, Lark L.Lohmann, VolkerSong, JiuzhouCOHRS, RANDALLLokensgard, James RSönnerborg, AndersCoiras, MayteLópez, DanielSorokulova, IrynaColberg-Poley, AnamarisLópez-Ferber, MiguelSozhamannan, ShanmugaConnolly-Andersen, Anne-MarieLossius, AndreasSpencer, Juliet V.Conrad, NicholasLuciani, FabioSpilki, FernandoConzelmann, KlausLuftig, MicahStamminger, ThomasConzelmann, Karl-KlausLukac, DavidStanton, RichardCook, Atlanta G.Lukashevich, IgorStanton, Robert C.Cory, JenyLundstrom, KennethStanway, GlynCosset, François-LoïcLünemann, Jan D.Steinmann, EikeCoutts, Robert H. A.Luo, ShuhongStenglein, MarkCoyne, CarolynLuo, DahaiStephens, EdwardCraigie, RobertMa, WenjunStern, PeterCroker, BenMacchi, BeatriceSteven, Alasdair C.Crump, ColinMacdonald, AndrewStewart, MeredithCsorba, TiborMachamer, CarolynStice, Steve L.Dąbrowska, KrystynaMacLachlan, JamesStojdl, DavidDainat, BenjaminMadan, VanesaStoltzfus, C. MartinDalianis, TinaMaecker, HoldenStonehouse, NicolaDalmo, Roy AMaekawa, ShinyaStoye, JonathanDavenport, MilesMagae, JunjiStrick, ReinerDawson, Christopher W.Mager, DixieStrong, JimDay, Patricia M.Mahony, Timothy JohnStrong, JamesDay, James MichaelMakinen, KristiinaSui, GuangchaoDe Francesco, RaffaeleMaldarelli, FrankSullivan, Deborah E.de Graaf, DanielMameli, GiuseppeSullivan, ChrisDe La Torre, Juan CarlosMancini, NicasioSullivan, Matthew B.De Los Santos, TeresaMarc, DanielSun, Bingde Miranda, JoachimMarcello, AlessandroSun, Rende Motes, Carlos MaluquerMarchini, AntonioSusi, Petride Pablo, PedroMarcotrigiano, JosephSuzuki, JonDe Silva, Aravinda M.Maria Isabel, Sola GurpeguiSuzuki, TohruDebarbieux, LaurentMaria Vaira, AnnaSvitkin, YuriDebyser, ZegerMarjomäki, VarpuSwamy, Manjunath N.DeFilippis, Victor R.Marjuki, HenjuSwanson, ChadDelang, LeenMarkov, Peter V.Swigon, DavidDelmas, BernardMarkovitz, DavidSwitzer, WilliamDenner, JoachimMarques, JoaoTagaya, YutakaDeom, CarlMarsh, MarkTakeda, ShigekiDesbiez, CécileMarshall, John A.Takegami, TsutomuDhurandhar, NikhilMartinez, OsvaldoTakeuchi, YasuhiroDiallo, Jean-SimonMartínez, IsidoroTakimoto, ToruDiamond, Don J.Martínez-Salas, EncarnaciónTamori, AkihiroDíaz Pendón, Juan AntonioMartinez-Sobrio, LuisTanaka, YuetsuDiedrich, SabineMarzano, ShinyiTang, HengliDiefenbach, Russell J.Masson, JeanTedbury, PhilipDietzgen, Ralf G.Matranga, ChrisTee, Kok-KengDiez, JuanaMatsukura, KeiichiroTempesta, MariaDimaio, DanielMatthews, StephenTerajima, MasanoriDimitrov, DimiterMaury, WendyTesh, Robert B.Dobner, ThomasMayer, JensTheilmann, DavidDobrovolny, Hana M.Mazurov, Dmitriy VThéry, ClotildeDolei, AntoninaMcBride, AlisonThézé, JulienDolnik, OlgaMcCance, DennisThorne, Stephen H.Dominguez, SamuelMcCauley, John W.Tietjen, IanDonato, CelesteMcclure, MyraTikoo, Suresh K.Doorbar, JMcCormick, CraigTommasino, MassimoDoublet, VincentMcElroy, AnitaTommassino, MassimoDräger, CarolinMcFadden, GrantTorres, JaumeDreux, MarleneMcInerney, GeraldTravé, GillesDrexler, Jan FelixMcinnes, ColinTrobridge, Grant D.Dropulić, BoroMcLaughlin-Drubin, MargaretTsai, BillyDrosten, ChristianMcminn, Peter C.Tsai, C.H.Drulis-Kawa, ZuzannaMcnamara, JamesTsukiyama-Kohara, KyokoDubuisson, JeanMcTaggart, Seanna JTurnbull, Matthew W.Duerksen-Hughes, Penelope J.Medina, Rafael A.Ueda, KojiDvorak, CherylMeeus, IvanUeda, KeijiEdgar, JamesMeijer, AdamUprichard, Susan LEichwald, CatherineMeins, Frederick Jr.Urcelay, ElenaEl Zowalaty, MohamedMelero, José A.Vähä-Koskela, MarkusElbeaino, TouficMellors, John W.Vahidnia, FarnazElder, John H.Mendoza-Cano, FernandoValles, Steven M.Elshabrawy, HatemMenendez-Arias, LuisVallinoto, AntonioEmerson, GinnyMeneses, Patricio I.van den Ham, Henk-JanEnjuanes, LuisMercer, AndrewVan Den Hoogen, Bernadette G.Enquist, Lynn W.Mercer, JasonVan Den Hurk, AndrewEnsser, ArminMergia, AyalewVan der Kuyl, AntoinetteEriksson, KristinaMerits, Andresvan der Poel, WimErlandson, MartinMeseda, ClementVan Duyne, RachelErrington-Mais, FionaMetcalf, Jordan P.Van Etten, JamesEssani, KarimMetzner, KarinVan Grevenynghe, JulienEtebari, KayvanMeyers, Gregorvan Hoek, B.Evans, MatthewMichiels, Thomasvan Lier, RenéEverett, Roger D.Millard, AndrewVan Raaij, MarkFavier, Anne-LaureMiller, Sara EVan Sinderen, DouweFederico, MaurizioMiller, StefanVander Hoek, LiaFeng, PinghuiMiller, GeorgeVaneechoutte, MarioFerrante, PasqualeMiller, E. KathrinVanham, GuidoFerrari, GuidoMinsky, AbrahamVarzakas, TheodorosFielding, Burtram C.Mintz, PaulVassilopoulos, GeorgeFilippov, ValeryMlotshwa, SizolwenkosiVeluthambi, KaruppananFillat, CristinaMochizuki, TomofumiVenuti, AldoFinke, StefanMonier, AdamVerbeek, MartinFischer, Wolfgang B.Moody, CaryVerdaguer, NuriaFisher, ChrisMoore, Sean D.Verheije, HélèneFleming, SteveMoreau, HervéVerheyen, JensFlenniken, MichelleMorgan, IainVerma, Subhash CFlint, JaneMori, SeiichiroVidal, SilviaFlodström-Tullberg, MalinMorrical, Scott W.Vieillard, VincentFlores, RicardoMorrison, RichardVilches, CarlosFlores, Eduardo FMorrison, Trudy GVillalain, JoseFlorin, LuiseMortreux, FranckVisseaux, BenoitFoley, Brian T.Morya, VivekVitour, DamienForlenza, MariaMoseley, GregoryVlak, Just M.Fouts, Derrick EMoss, BernardVogel, FranziskaFreed, Eric O.Mothes, Walther H.Volchkova, ValentinaFrensing, TimoMourier, Tobiasvon Hahn, ThomasFriedland, RobertMueller, ThomasWaheed, Abdul A.Friedman, Gregory K.Mukhopadhyay, SuchetanaWainberg, MarkFrieman, MBMulder, LubbertusWakimoto, HiroakiFrisk, GunMulkidjanian, ArmenWalker, ThomasFu, ZhenMuller, Claude P.Wan, YonghongFuentes, AlejandroMünch, JanWanasen, NanchayaFukushi, HidetoMunster, VincentWang, QianGallagher, TomMunster, Vincent J.Wang, ShuGallay, PhillipMurata, TakayukiWang, JieruGanges, LlilianneMurphy, LitaWang, Robert Y.L.Gao, FengMussolino, ClaudioWang, Chi-YoungGara, NaveenMuylkens, BenoîtWang, Jen-renGarcía, MaricarmenMymryk, JosephWang, SongjieGarcia, PilarN. Frick, DavidWang, YingGarcia-Estañ, Luis PerezNaesens, LieveWang, Ching-hoGarozzo, AdrianaNagata, NoriyoWang, Han-ChingGarrote, José Ignacio NúñezNagy, Peter D.Wang, Pei GangGatignol, AnneNájera, IsabelWarfield, Kelly L.Geisbert, thomas wNakahara, Kenji S.Waris, GulamGenersch, ElkeNekhai, SergeiWatanabe, ToshikiGeorgel, PhilippeNelson, KenradWeber, IreneGessain, AntoineNetherton, Christopher L.Węgrzyn, GrzegorzGhany, MarcNettelbeck, Dirk M.Wei, XierongGhose, RanajeetNg, Lisa F.P.Wei, YangdouGiam, JoeNicoletti, LoredanaWeinman, StevenGifford, Robert J.Nieto, AmeliaWeissenhorn, WinfriedGilbert, CarolineNilsson, AndersWeitzman, MatthewGilbert, ClémentNisole, SebastienWeller, SandraGillet, NicolasNitsche, AndreasWhite, KirstenGillet, LaurentNoda, HiroakiWhitehurst, ChristopherGisder, SebastianNolasco, GustavoWiebe, Matthew S.Goffard, AnneNorder, HeléneWileman, TomGoldmana, Ellen R.Novella, IsabelWilen, CraigGoldsmith, CynthiaNowotny, NorbertWilhelm, BarbaraGolovanov, Alexander P.Nunes, Márcio R. T.Willems, LucGong, PengNuttall, Patricia AWilliams, TrevorGoodrum, FeliciaO'connor, David H.Wills, JohnGorbalenya, Alexander E.Obar, Joshua J.Wilson, VanGorini Da Veiga, AnaOberste, StevenWimley, William C.Gossner, CelineObrępalska-Stęplowska, AleksandraWimmer, EckardGötte, MatthiasOhlmann, ThéophileWitcher, MichaelGraham, SheilaOhshima, TakayukiWittmann, JürgenGramberg, ThomasOkazaki, KatsunoriWong, Sek ManGrandgenett, Duane P.Okino, Cintia HiromiWood, Henry M.Grandvaux, NathalieOlinger, GeneWorgall, StefanGrattagliano, IgnazioOliver, StefanWorkenhe, SamuelGrdzelishvili, Valery Z.Olson, KennethWu, YuntaoGreber, Urs F.Ono, AkiraXi, ZhiyongGreen, Patrick L.Ornelles, DavidYaegashi, HajimeGregoire, MarcOrtego, JavierYamanaka, AtsushiGriffiths, PaulOshitani, HiroshiYamauchi, YoheiGriffiths, AnthonyOw, ThomasYang, ChinglaiGronenborn, BrunoOzbun, MichelleYang, ChunfuGrzywacz, DavidPagano, Joseph S.Yang, DechengGuillon, ChristophePager, CaraYang, FengGummuluru, SuryaramPalamara, Anna TeresaYedavalli, VenkatGundogdu, OzanPalese, PeterYeh, Shyi-Dong Guo, Z. ShengPalmarini, MassimoYonekura, KentaroGupta, RashmiPaludan, Søren R.Yu, Xiao-FangGupte, SachinPan, QiuweiYuan, WeimingGustin, Jean K.Panchal, Rekha G.Zakhartchouk, AlexanderGustin, KurtPandha, HardevZamarin, DmitriyHaas, Juergen G.Papic, NevenZell, RolandHajimorad, RezaPappu, HanuZeltins, AndrisHakami, RaminParadkar, PrasadZerbini, Francisco MuriloHall, RoyParent, LeslieZhang, YanjinHan, Guan-zhuParent, Kristin N.Zhang, Lian-fengHarris, KatharineParish, Joanna L.Zhang, JianqiangHarrison, RobertPathak, VinayZhang, QijingHarrod, RobertPatil, BasavaprabhuZheng, Yong-HuiHartshorn, KevanPattnaik, AsitZhou, PengHayward, S. DianePatton, JohnZur Hausen, H.He, MingliangPeden, Keith


